# ﻿Descriptions of two new flightless species of *Pseudocsikia* Schimmel & Platia (Coleoptera, Elateridae, Dimini) from Taiwan Island, China, with a definition of the *formosana* species-group

**DOI:** 10.3897/zookeys.1103.84015

**Published:** 2022-06-01

**Authors:** Lu Qiu, Robin Kundrata

**Affiliations:** 1 Engineering Research Center for Forest and Grassland Disaster Prevention and Reduction, Mianyang Normal University, Mianxing West Road, 621000, Mianyang, China Mianyang Normal University Mianyang China; 2 Department of Zoology, Faculty of Science, Palacky University, 17. listopadu 50, 77146, Olomouc, Czech Republic Palacky University Olomouc Czech Republic

**Keywords:** China, Dendrometrinae, Elateroidea, flightlessness, new species, Taiwan

## Abstract

Two new flightless click beetle species, *Pseudocsikiachoui***sp. nov.** and *Pseudocsikiachanjuan***sp. nov.**, are described and illustrated from Taiwan, China. Their habitus and diagnostic characters are illustrated. The two species most resemble *P.formosana*, which is endemic to Taiwan, by the strongly protruding pronotal anterior angles accompanied by pits and the shape of aedeagus. They can be all grouped as the *P.formosana*-species group. A key to the species of the *P.formosana*-species group and an updated checklist of Chinese *Pseudocsikia* with supplementary notes on type localities are provided. The discovery of two new species highlights the potential species-richness of the flightless click-beetles on Taiwan Island.

## ﻿Introduction

*Pseudocsikia* Schimmel & Platia, 1991 (Elateridae, Dendrometrinae, Dimini) is a small genus of click-beetles known from China, Myanmar, Laos, India, and Nepal (Kundrata et al. 2018). Schimmel and Platia (1991) established this genus for two species, *P.rustica* Schimmel & Platia, 1991 and *P.laticollis* Schimmel & Platia, 1991, both from Nepal. Later, Schimmel (1993, 1996a) transferred five species from other genera to *Pseudocsikia*: *Csikiaformosana* Ôhira, 1972 from Taiwan Island, China, *Csikiamanipurensis* Schimmel & Platia, 1992 from India, *Peniabirmanica* Candèze, 1888 and *Peniafausta* Candèze, 1888 from Myanmar, and *Peniadorsalis* Fleutiaux, 1936 from Laos. Schimmel (1996b, 2006) described three more species in the genus, *Pseudocsikiagaoligongshana* Schimmel, 1996 and *P.turnai* Schimmel, 2006 from mainland China, and *P.phongsalyana* Schimmel, 2006 from Laos. Hence, 10 species are currently included in this genus (Kundrata et al. 2018).

Although several works (Schimmel and Platia 1991; Schimmel 1993, 1996a, 1996b, 2006) contributed to the knowledge of *Pseudocsikia*, the generic concept remains been vague. Only Schimmel and Platia (1991) and Schimmel (1996a) fundamentally defined the genus. According to these works, *Pseudocsikia* can be distinguished among Dimini by the following combination of characters: pronotum widest near the middle, as wide as or wider than elytra, hind angles long, pointed and extended straight toward the base of elytra, elytra short, arched laterally in dorsal view, tarsomeres III–IV lobate ventrally, metacoxal plate covering near half or more of metatrochanter.

Here, we describe two new *Pseudocsikia* species from Taiwan Island, China. Both species are flightless and share many similar characters with *P.formosana*, which is also known from Taiwan. A species group is defined to include these three species. The discovery of these species suggests that the flightless Dimini in China can be species-rich not only in the mainland habitats but also on continental islands.

## ﻿Material and methods

**Figure 1.**
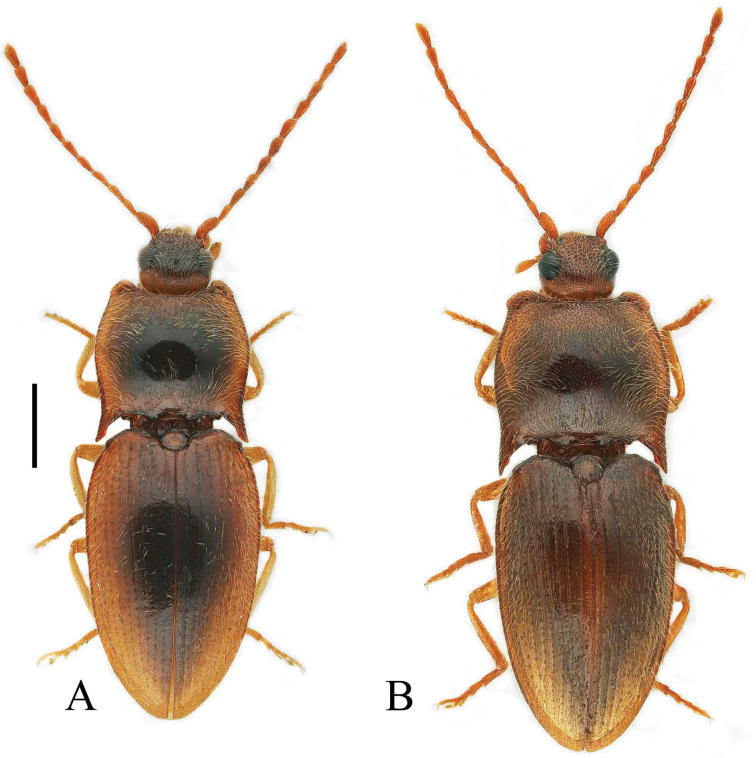
Habitus of *Pseudocsikia* spp. **A***Pseudocsikiachoui* sp. nov., male holotype, dorsal view **B***Pseudocsikiachanjuan* sp. nov., male holotype, dorsal view. Scale bar: 1.0 mm.

**Figure 2.**
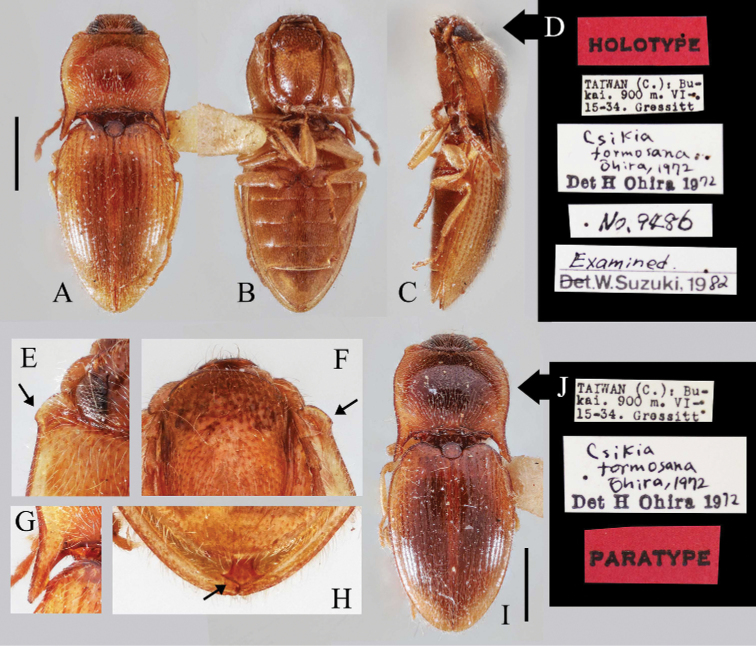
Habitus and characters of *Pseudocsikiaformosana* (Ôhira, 1972) **A–H** male holotype **A** habitus, dorsal view **B** habitus, ventral view **C** habitus, lateral view **D** labels **E** anterior protrusion of pronotum (indicated by an arrow), dorsal view **F** anterior protrusion of hypomeron (indicated by an arrow), ventral view **G** posterior angle of pronotum, dorsal view **H** abdominal tip, ventral view (arrow indicates the apex of median lobe) **I, J** paratype of an unknown sex **I** habitus, dorsal view **J** labels. Scale bars: 1.0 mm (**A–C, I**); **E–H** not to scale. All photos provided by Jeremy Frank (BPBM).

**Figure 3.**
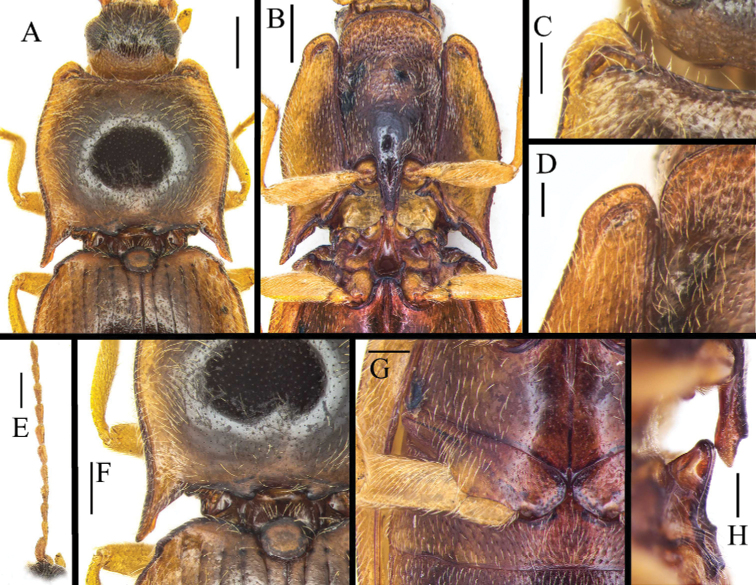
Characters of *Pseudocsikiachoui* sp. nov., male, holotype **A** pronorum, dorsal view **B** pro- and mesothorax, ventral view **C** anterior protrusion of pronotum, dorsal view **D** anterior protrusion of hypomeron, ventral view **E** antenna **F** posterior angle of pronotum, dorsal view **G** metacoxal plate, ventral view **H** prosternal process, lateral view. Scale bars: 0.5 mm (**A, B, E, F**); 0.2 mm (**C, D, G, H**).

**Figure 4.**
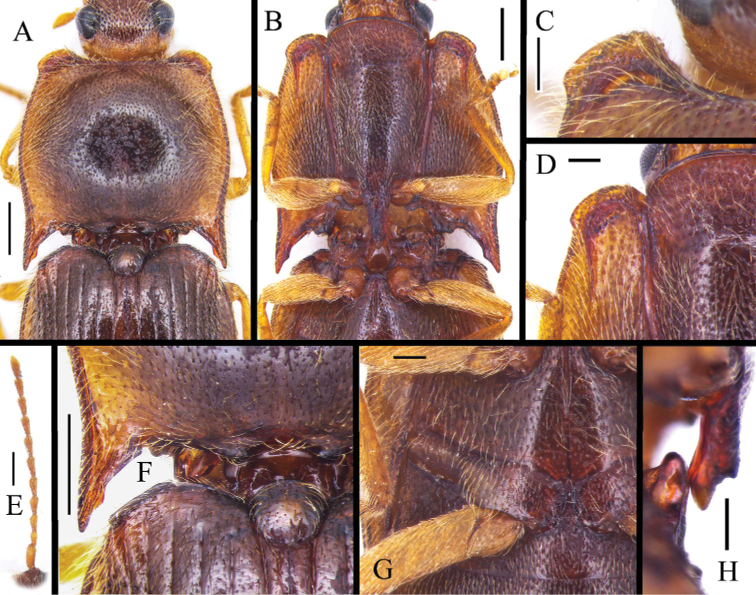
Characters of *Pseudocsikiachanjuan* sp. nov., male, holotype **A** pronorum, dorsal view **B** pro- and mesothorax, ventral view **C** anterior protrusion of pronotum, dorsal view **D** anterior protrusion of hypomeron, ventral view **E** antenna **F** posterior angle of pronotum, dorsal view **G** metacoxal plate, ventral view **H** prosternal process, lateral view. Scale bars: 0.5 mm (**A, B, E, F**); 0.2 mm (**C, D, G, H**).

**Figure 5.**
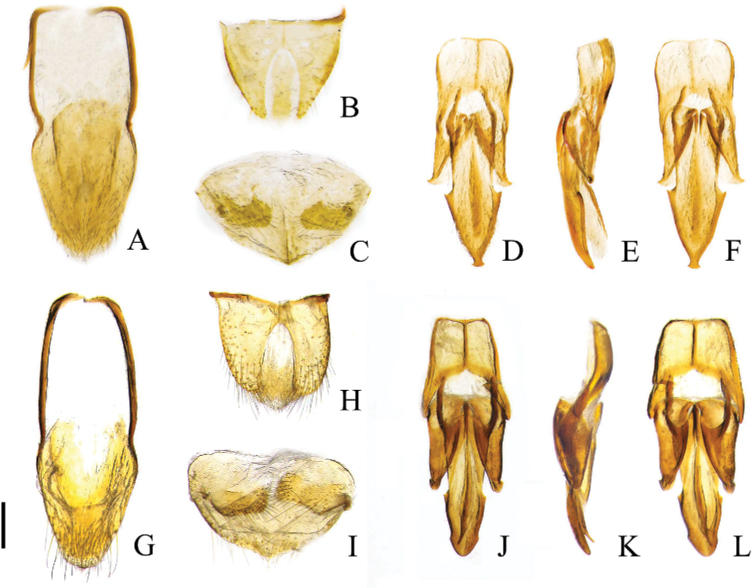
Characters of *Pseudocsikia* spp. **A–F***Pseudocsikiachoui* sp. nov., male holotype **G–L***Pseudocsikiachanjuan* sp. nov., male, holotype **A, G** abdominal sternite IX, dorsal view **B, H** abdominal tergites IX–X, dorsal view **C, I** abdominal sternite VIII and tergite VIII, ventral view **D, J** aedeagus, ventral view **E, K** aedeagus, lateral view **F, L** aedeagus, dorsal view. Scale bar: 0.2 mm.

The specimens were softened in hot water, and genital segments were excised and dissected after treatment in 80 °C 10% KOH for 10 min. Habitus images were photographed using a Canon EOS RP + Mount Adapter EF-EOS R + a Laowa 25 mm F2.8 2.5–5× Ultra Macro Lens (for Canon EF); diagnostic characters were made using a Leica M205A stereomicroscope and a Leica DFC 550. All figures (Figs 1–5) were modified in Adobe Photoshop CC 2019. Body length was measured from the anterior margin of the head to the apex of the elytra, pronotal length was measured at midline, pronotal width was measured both at the widest point and between hind angles, and body width was measured at the widest place of the elytra. The generic concept of *Pseudocsikia* follows Schimmel and Platia (1991) and Schimmel (1996a). The holotypes of the new species are deposited in the Invertebrate Collection of Mianyang Normal University, Mianyang, Sichuan, China (**MYNU**). The holotype and one paratype of *P.formosana* are deposited in the Bernice Pauahi Bishop Museum, Honolulu, Hawaii, USA (**BPBM**). The collecting data is quoted verbatim (in Chinese) in quotation marks. Translation of the data, as well as additional information, is given in square brackets.

## ﻿Systematics

### ﻿Genus Pseudocsikia

Taxon classificationAnimaliaColeopteraElateridae

Schimmel & Platia, 1991

F8B9498E-2613-5830-A4EB-ABBDCD41D3FF

Pseudocsikia Schimmel & Platia, 1991: 357 (original description); Schimmel 1996a: 203 (revision); Cate et al. 2007: 186 (catalogue); Kundrata et al. 2018: 65 (catalogue).

#### Type species.

*Pseudocsikiarustica* Schimmel & Platia, 1991.

### *Pseudocsikiaformosana*-species group, here defined

**Diagnosis.** Anterior angles of pronotum lateroapically protruded, with anterior edge of pronotum mesally concave in dorsal view. Each protrusion with sides almost parallel in dorsal view, with concavity laterally and a deep pit at basal portion (Figs 2E, 3C, 4C). Hypomeron with long carination parallel to pronotosternal suture and following curved outline of anterior protrusion of hypomeron, with small pit on the inside edge (Figs 2F, 3D, 4D). Male genitalia (Fig. 5) with robust median lobe, distal half enlarged, variously shaped. Parameres short, stout, about half as long as median lobe. Phallobase with thickened outlines, and medially with longitudinal line.

**Remarks.** The *P.formosana*-species group is known only from Taiwan and is possibly endemic. All three species are easily distinguished from congeners by the structure of anterior angles of pronotum, which are stoutly protruded, with a abrupt concavity laterally, and with a large deep pit at the basal portion of each protrusion. Such characters are not present in the type species of *Pseudocsikia*, *P.rustica* (Schimmel and Platia 1991), or any other *Pseudocsikia* species (Schimmel and Platia 1991; Schimmel 1993, 1996a, 1996b, 2006). Within Dimini, protruded anterior angles of pronotum can be found in several other Dimini, like for example, *Platiana* Schimmel, 1993 or most species of *Parapenia* Suzuki, 1982 (Suzuki 1982; Schimmel 1996a), but the protrusions in these species are more or less gradually narrowed to a point, and either with larger pits located anteriorly or with only small, shallow pits. These unique characters of Taiwanese *Pseudocsikia* suggest a possible need for a new genus to accommodate them. However, they should be kept in *Pseudocsikia* until evidence from a detailed revision or phylogeny is available.

**Species included.***Pseudocsikiaformosana* (Ôhira, 1972), *P.choui* sp. nov., *P.chanjuan* sp. nov.

**Distribution.** China (Taiwan).

### ﻿ Pseudocsikia choui sp. nov.

Taxon classificationAnimaliaColeopteraElateridae

1E8BE719-5A7D-5507-812A-131B5161B2AA

http://zoobank.org/9AD84855-59B9-4C47-9734-FC6A2AAC723E

Figs 1A , 3A–H , 5A–F

#### Type material.

***Holotype***, male, “2017.IX.13,台湾嘉义县阿里山二万坪, 2000m, 周文一” [Erwanping, Mount Alishan, Chiayi County, Taiwan, 2000 m, 13.IX.2017, Wen-I Chou leg.], “*Pseudocsikiachoui* sp. nov. 周氏伪斯叩甲 HOLOTYPE des. Qiu et Kundrata 2022” (MYNU).

#### Diagnosis.

Head, pronotum, and elytra dark brown, with paler lateral portions of pronotum and elytra, legs yellow (Fig. 1A). Antennomere II subequal in length to antennomere III. Pronotum (Fig. 3A) smooth, with sparse punctures (intervals usually equal to 4–6 puncture diameters). Anterior angle of pronotum with apex of protrusion closer to inner angle. Posterior angles divergent. Metaventrite sparsely punctate, intervals between punctures on average subequal to 2–5 puncture diameters. Metacoxal plate (Fig. 3G) enlarged mesally, sparsely punctate. Tergite IX (Fig. 5B) subtriangular, with two narrow lobes. Aedeagus with median lobe with acute lateral projetions near midlength, narrowed to apex, apex blunt with small acute lateral projections. Paramere with apex pointed and projecting laterad. Phallobase with basal angles rounded (Fig. 5D).

#### Comparison.

This species superficially resembles *P.fomosana* by the pale coloration of its pronotum and elytral sides and by the sparse punctures of pronotum, but it can be easily distinguished from the latter by the larger body length (5.9 mm, while 4.0 mm in *P.formosana*), darker coloration of pronotum and elytra medially, more forwardly protruded anterior angles of pronotum (pointing more outward in the pronotum of *P.formosana*), and shorter and more divergent posterior angle of pronotum (longer, more robust and nearly straight in *P.formosana*). The shape of aedeagus also readily differentiates these two species. Based on the illustration of Ôhira (1972: fig. 3), the distal half of the median lobe of *P.formosana* has four large acute processes laterally, and its apex is somewhat rectangular; and the paramere of *P.formosana* has the apex rounded and slightly outward.

#### Description

**(male holotype).** Body smooth, surface covered with curved, semi-erect, and moderately long pubescence. Body length 5.9 mm; width 2.2 mm; antenna length 3.0 mm; pronotum length 1.4 mm, pronotum width 1.9 mm (measured at posterior angles), elytra length 3.5 mm.

Body brown, pubescence yellow (Fig. 1A). Head dark brown, antennae yellowish brown, labrum and mandibles brown, remaining mouthparts yellowish brown. Pronotum dark brown centrally; lateral, anterior and hind portions, and hind angles yellowish brown, with darker outlined margins. Scutellar shield brown, with dark outlined margins, especially anteriorly. Elytra dark brown centrally, yellowish brown laterally; yellowish-brown portions gradually lightened toward apices, basal margins of elytra dark outlined. Underside reddish brown, prosternum darker than hypomeron, sternites VI–VII and legs yellow.

Head including eyes 0.5 times as wide as pronotum. Supra-antennal carinae short, directed mesad and fading medially so that median portion of frontoclypeus is not formed by sharp carina; frontoclypeus overhanging base of labrum in lateral view. Head surface sparsely punctate; punctures small, intervals between punctures mostly equal 2–3 puncture diameters. Maxillary palpus with palpomere III longer than wide. Antenna (Fig. 3E) surpassing hind angle of pronotum by about one antennomere; scape robust and longest, remaining antennomeres subequal in length; ultimate antennomere obliquely truncate, with apex rounded.

Pronotum (Fig. 3A) wider than long (measured at midline), widest near middle. In lateral view, pronotum convex. Anterior angles of pronotum protruding (Fig. 3C); protrusion of anterior angle subquadrate, inner angle more protruded than outer angle, posterior part of protrusion with deep, crescent-shape pit. Lateral margins of pronotum arched medially, sides near middle narrowing anteriad and posteriad, anteriorly narrowing more sharply than posteriorly; posterior angles (Fig. 3F) long, slightly divergent, apical portion of posterior angle slightly enlarged, then narrowed, apex blunt. Disc of pronotum sparsely covered with small, shallow punctures; intervals between punctures on average subequal to four to six puncture diameters; interstices smooth. Pubescence mostly directed outwards; basal portion directed anteriorly.

Hypomeron (Fig. 3B) more densely punctate than pronotum, punctures small and shallow, intervals between punctures on average subequal to 3–4 puncture diameters; apex of hypomeron strongly protruded, margin wrinkled. Pronotosternal sutures nearly straight, anterior excavation wide, long carination paralleled with suture from base of hypomeron and reaching anterior protrusion of hypomeron, forming hook-shaped carination anteriorly, end of the carination slightly extending backwards, with a small pit partly enclosed by curving hook of carination (Fig. 3D). Prosternum (Fig. 3B) including prosternal process about 2.00 times as long as wide; chin piece with large, dense punctures, intervals between punctures approximately one puncture diameter; punctures in remaining area sparser and smaller, intervals between punctures 3–6 puncture diameters, punctures on prosternal process sparse, small. Prosternal process (Fig. 3H) with ventral surface horizontal in lateral view, with elongate notch ventroapically, roundly enlarged dorsoapically.

Scutellar shield (Fig. 3F) suboval, about 1.2 times as wide as long; anterior margin rounded, posterior margin slightly pointed. Mesoventrite (Fig. 3B) with deep procoxal rests. Mesoventral process elevated, hind margin wide. Mesanepisternum with large, curved lateral extensions of procoxal rests. Metaventrite medially with sparse punctures, intervals between punctures on average subequal to 3–5 puncture diameters. Anterior portion of discrimen with sharp groove, occupying half-length of metaventrite. Metacoxal plate enlarged inward, narrowed laterad (Fig. 3G), surface with very sparse punctures.

Elytra (Fig. 1A) elongate, together 1.6 times as long as wide, widest at 1/3 of their length from base. Humeri (Fig. 3A, F) elevated. Sides from humeri roundly widened to 1/3 of elytral length in dorsal view, then gradually narrowed towards apices; apices slightly independently rounded. Elytral striae shallow, formed by lines of small punctures, intervals between punctures in stria on average subequal to 2–3 puncture diameters. Interstriae flat, smooth, with some micropunctures. Hind wings absent. Abdomen with ventrites more densely punctate than pronotum, intervals between punctures on average subequal to 2–3 puncture diameters; pubescence directed backwards. Apical ventrite with blunt apex. Tergite VIII (Fig. 5C) subtriangular, 1.6 times as long as wide, distal margin pointed medially, apically covered with sparse pubescence. Sternite VIII with two dark colored lobes, shape as Fig. 5C, with long setae, remaining portion membranous. Tergite IX (Fig. 5B) subtriangular, 1.3 times as long as wide, medially deeply emarginate; two lobes elongate, lateral sides with long setae; tergite X (Fig. 5B) membranous, exceeding apices of lobes of tergite IX. Sternite IX (Fig. 5A) slightly stout, 2.7 times as long as wide, apically widely rounded and setose.

***Aedeagus*** (Fig. 5D–F) with robust median lobe, two times as long as one paramere; distal half of median lobe arrow-shaped, apex with small protrusion, apex blunt, laterally with small acute projections; long, needle-like sclerite present on ventral side of median lobe. Paramere stout, reaching half of median lobe; apex pointed outward. Phallobase subquadrate, margins thickened, medially with longitudinal thickened line, basal angles rounded.

**Female.** Unknown.

#### Immature stages.

Unknown.

#### Distribution.

China: Taiwan (Chiayi).

#### Etymology.

The specific patronymic epithet is dedicated to Dr Wen-I Chou (Taiwan, China), the collector of the holotype.

### ﻿ Pseudocsikia chanjuan sp. nov.

Taxon classificationAnimaliaColeopteraElateridae

16003A22-D969-5423-B977-AB956F641787

http://zoobank.org/835DC83E-6E39-437D-8A81-7C78F81757A9

Figs 1B , 4A–H , 5G–L

#### Type material.

***Holotype***, male, “2017.IX.16, 台湾台东县金峰乡太麻里山, 1300 m, 周文一” [Mount Taimalishan, Taitung County, Taiwan, 1300 m, 16.IX.2017, Wen-I Chou leg.], “*Pseudocsikiachanjuan* sp. nov. 婵娟伪斯叩甲 HOLOTYPE des. Qiu et Kundrata 2022” (MYNU).

#### Diagnosis.

Pronotum and elytra almost unicolored brown, but with paler apices of elytra and lateral margins of pronotum (in dry specimen condition), legs yellow (Fig. 1B). Antennomere II shorter than the length of antennomere III. Pronotum (Fig. 4A) with dense punctures (intervals usually subequal to 2–4 puncture diameters). Anterior angle of pronotum with the protrusion outward at outer angle. Posterior angle straight. Metaventrite densely punctate, intervals between punctures on average subequal to 2–3 puncture diameters. Metacoxal plate (Fig. 4G) short internally, surface densely covered with punctures. Tergite IX (Fig. 5H) suboval, with two robust lobes. Median lobe with small lateral pointed process near midlength, apical portion rounded and enlarged. Paramere with rounded apex and small process subapically. Phallobase subtrapezoidal, with slightly pointed basal angles (Fig. 5J).

#### Comparison.

This species can be distinguished from *P.formosana* and *P.choui* sp. nov. by the denser punctures of pronotum and larger body size (6.3 mm versus 4.0–5.9 mm). This new species can be further distinguished from *P.choui* sp. nov. by the more outwardly protruded anterior angles of pronotum, and the larger and straight posterior angle of pronotum. The shape of aedeagus also differs from these. The median lobe of *P.chanjuan* sp. nov. has a slightly enlarged and rounded apex and two small acute processes laterally near midlength, its paramere is rounded at apex but with small process subapically, and the phallobase is less rounded basally than those of the other two species.

#### Description

**(male holotype).** Body smooth, surface covered with curved, semi-erect, and moderately long pubescence. Body length 6.3 mm; width 2.3 mm; antenna length 3.3 mm; pronotum length 1.7 mm, width 2.1 mm (measured at hind angles), elytra length 3.7 mm.

Body generally brown, pubescence yellow (Fig. 1B). Head (including antennae and mouthparts), pronotum, elytra (except apical portions), underside (except last two sternites and lateral portion of abdomen) brown. Pronotum with paler lateral margins (in dry condition). Legs, apical portion of elytra, the last two sternites, and lateral portion of abdomen yellowish brown.

Head including eyes 0.5 times as wide as pronotum. Supra-antennal carinae short, directed mesad and fading medially so that median portion of frontoclypeus is not formed by sharp carina; frontoclypeus overhanging base of labrum in lateral view. Head surface with intervals between punctures mostly equal 1–2 puncture diameters. Maxillary palpus with palpomere III longer than wide. Antenna (Fig. 4E) simple, surpassing hind angle of pronotum about 1½ antennomeres; scape robust and longest, antennomere II shortest, antennomere III longer than antennomere II, antennomeres IV–X subequal in length, ratio of antennomeres II–IV and XI = 1: 1.1: 1.3: 1.5, ultimate antennomere tapered apically, apex pointed.

Pronotum (Fig. 4A) large, subquadrate, wider than long (measured at midlines), widest near middle. In lateral view, pronotum convex. Anterior angles (Fig. 4C) of pronotum protruded, protrusion of anterior angle subquadrate, inner angle protruded almost same degree as outer angle; prostrusion with deep, narrow and curved gap. Lateral margins of pronotum roundly arched medially, sides near middle more or less evenly narrowing anterad and posterad in similar degree, posterior angle (Fig. 4F) straight, less divergent, pointing straightly toward elytra, apex blunt, inner margin with small protrusion. Disc of pronotum densely covered with small, deep punctures; intervals between punctures on average subequal to 2–4 puncture diameters; interstices smooth. Pubescence directed outwards; basal portion directed forwards.

Hypomeron (Fig. 4B) more densely punctate than pronotum, punctures moderate and deep, intervals between punctures on average subequal to 1–2 puncture diameters, apex of hypomeron strongly protruded, margin wrinkled. Pronotosternal sutures nearly straight, anterior excavation deep and narrow; long carination paralleled with suture from base of hypomeron and reaching anterior protrusion of hypomeron, forming elongate U-shaped carination anteriorly; end of the carination extending backwards, with a straight, elongate pit partly enclosed by curving hook of carination (Fig. 4D). Prosternum (Fig. 4B) including prosternal process 2.2 times as long as wide; chin piece with dense and large punctures, intervals between punctures on average subequal to half to one puncture diameter; punctures in remaining area slightly sparser and smaller, intervals between punctures on average subequal to 1–2 puncture diameters. Prosternal process (Fig. 4H) ventrally straight in lateral view, ventroapically with notch; small process in notch acutely enlarged dorsoapically.

Scutellar shield (Fig. 4A, F) suboval, about 1.2 times as wide as long; anterior margin rounded, posterior margin slightly pointed.

Mesoventrite (Fig. 4B) with procoxal rests. Mesoventral process elevated, hind margin narrow. Mesanepisternum with large, curved lateral extensions of procoxal rests. Metaventrite medially with dense punctures, intervals between punctures on average subequal to 2–3 puncture diameters. Anterior portion of discrimen with needle-like groove, occupying half-length of metaventrite. Metacoxal plate enlarged inward, strongly reduced outward (Fig. 4G), surface densely punctate.

Elytra (Fig. 1B) elongate, together 1.7 times as long as wide, widest at 1/3 of their length from base. Humeri (Fig. 4A, F) elevated, sides from humeri roundly widened to 1/3 of elytral length, then gradually narrowed towards apices; apices slightly independently rounded. Elytral striae shallow, formed by lines of small punctures, intervals between punctures in stria on average subequal to 2–4 puncture diameters. Interstriae flat, smooth, with some micropunctures. Hind wings absent. Abdomen with ventrites more densely punctate than pronotum, intervals between punctures on average subequal to one puncture diameter; pubescence directed backwards. Apical ventrite with rounded apex. Tergite VIII (Fig. 5I) subtriangular, 1.7 times as long as wide, distal margin pointed medially, apically covered with sparse pubescence, basal angles rounded. Sternite VIII (Fig. 5I) with two dark-colored lobes, shape as Fig. 5I with long setae, remaining portion membranous. Tergite IX (Fig. 5H) semi-oval, 1.2 times as long as wide, medially deeply and widely emarginate; two lobes robust, lateral sides with long setae; tergite X (Fig. 5H) membranous, exceeded apices of lobes of tergite IX. Sternite IX (Fig. 5G) relatively narrow, 2.66 times as long as wide, apically truncate and setose.

Aedeagus (Fig. 5J–L) with robust median lobe, 1.7 times as long as one paramere; distal half of median lobe gradually narrowed to a rounded point, with one small pointed processes on each lateral side near midlength, apical portion enlarged, apex bluntly rounded; median lobe with long, needle-like ventral sclerite. Paramere stout, slightly exceeding half of median lobe; apex rounded, with small process subapically. Phallobase trapezoidal, margins thickened, medially with longitudinal thickened line, basal angles angled.

**Female.** Unknown.

#### Immature stages.

Unknown.

#### Distribution.

China: Taiwan (Taitung).

#### Etymology.

The specific epithet *Chanjuan* is derived from the Chinese 婵娟 [chán juān], which means “beauty”.

### ﻿ Pseudocsikia formosana

Taxon classificationAnimaliaColeopteraElateridae

(Ôhira, 1972)

93A30EAF-26ED-5558-96D7-C9A1E5BC09A3

Fig. 2A–J

Csikia formosana Ôhira, 1972: 8 (original description); Bouwer 1991: 57 (checklist); Jiang 1993: 146 (catalogue); Suzuki 1999: 120 (catalogue).
Pseudocsikia formosana (Ôhira, 1972): Schimmel 1993: 255 (new combination); Schimmel 1996a: 204 (diagnosis); Cate et al. 2007: 186 (catalogue); Kundrata et al. 2018: 66 (catalogue).

#### Type material.

***Holotype*** of *Csikiaformosana* Ôhira, 1972, male, “TAIWAN (C.): Bu-kai. 900 m. VI.15–34. Gressitt”, “Csikiaformosana Ôhira, 1972 Det H Ohira 1972”, “HOLOTYPE”, “No. 9486”, “Examined. Det. W. Suzuki, 1982” (BPBM). One ***paratype*** of *Csikiaformosana* Ôhira, 1972, sex unknown, “TAIWAN (C.): Bu-kai. 900 m. VI-15-34. Gressitt”, “Csikiaformosana Ohira, 1972 Det H Ohira 1972”, “PARATYPE” (BPBM).

#### Diagnosis.

(based on Ôhira 1972 and the figures of holotype and one paratype provided by BPBM). Body length 4.0 mm, width 1.7 mm (based on holotype; see Ôhira 1972). Body reddish brown (examined paratype darker than holotype; Fig. 2A–C, I), outer margins of pronotum and elytra, underside, and legs paler. Antenna exceeding posterior angle of pronotum by about apical two antennomeres (Ôhira 1972). Antennomere II slightly shorter than antennomere III (Ôhira 1972). Punctures on pronotum of moderate size, intervals between punctures mostly equal 3–5 puncture diameters, anterior protrusion of pronotum with apex laterad of center (Fig. 2E, F). Posterior angle (Fig. 2G) of pronotum long, straight and robust, apex blunt, inner margin with obtuse protrusion subapically.

Aedeagus with robust median lobe, twice as long as paramere; with large acute lateral processes near midlength, apex rectangular, with large subapical spines laterally (Fig. 2H) (needle-like ventral sclerite not mentioned in Ôhira 1972 and not observable in this study). Paramere stout, reaching half length of median lobe; apex rounded, slightly outwards. Phallobase subquadrate, margins thickened, medially with longitudinal thickened line, basal angles rounded (based on Ôhira 1972: fig. 3).

#### Distribution.

China: Taiwan (Nantou).

##### ﻿Checklist of *Pseudocsikia* species from China, with notes on their type localities

***Pseudocsikiaformosana* (Ôhira, 1972)**

**Chinese common name**: 台湾伪斯叩甲.

**Type locality**: “Bukai” (Ôhira 1972).

**Note**: Bukai is an old name of Fatyu [法治] (or Wuchieh [武界]) (Chu and Yamanaka 1973), Ren’ai township [仁爱乡], Nantou County [南投县], Taiwan.

***Pseudocsikiagaoligongshana* Schimmel, 1996**

**Chinese common name**: 高黎贡伪斯叩甲.

**Type locality**: “Yunnan, Gaoligongshan, 100 km westlich von Baoshan [100 km W of Baoshan]” (Schimmel 1996b).

**Note**: Gaoligongshan [高黎贡山] is an extensive mountain range lying on the border of Yunnan, China and Myanmar; the exact locality information of the holotype is unknown. However, according to the original paper, the holotype was collected 100 km west of Baoshan, which is near Tengchong.

***Pseudocsikiaturnai* Schimmel, 2006**

**Chinese common name**: 图氏伪斯叩甲.

**Type locality**: “China: Hubei-Provinz, 30 km nordostlich von Hefeng, Mulinzi [30 km NE of Hefeng, Mulinzi]” (Schimmel 2006).

**Note**: Mulinzi [木林子] is a nature reserve in Hefeng County [鹤峰县], Enshi City [恩施市], Hubei.

***Pseudocsikiachoui* Qiu & Kundrata, sp. nov.**

**Chinese common name**: 周氏伪斯叩甲.

**Type locality**: Erwanping, Mount Alishan, Chiayi County, Taiwan, 2000 m (this work).

***Pseudocsikiachanjuan* Qiu & Kundrata, sp. nov.**

**Chinese common name**: 婵娟伪斯叩甲.

**Type locality**: Mount Taimalishan, Taitung County, Taiwan, 1300 m (this work).

### ﻿Key to species of *Pseudocsikiaformosana*-species group

**Table:** 

1	Pronotum with anterior angles widely and strongly protruded with lateral concavity, with large pits at posterior part of protrusion in dorsal view	**2 (*P.formosana*-species group)**
–	Pronotum with anterior angles not protruded or simply, gradually and narrowly protruded, and without large pits at posterior part of protrusion if protruded	**other species of *Pseudocsikia***
2	Pronotum densely punctate, with average interval between punctures 2–4 puncture diameters (Fig. 4A); median lobe of aedeagus with one small pointed process on each side near midlength, apex simply enlarged, widely rounded, without acute projections (Fig. 5J)	***P.chanjuan* sp. nov.**
–	Pronotum sparsely punctate, with average intervals between punctures 3–6 puncture diameters (Figs 2A, 3A); median lobe of aedeagus with one large acute lateral projetion on each lateral side near midlength, apically or subapically with lateral acute projections	**3**
3	Median lobe of aedeagus with narrowed apical portion, apex additionally with blunt protrusion with small acute lateral projections; apex of paramere acute and pointing laterad (Fig. 5D)	***P.choui* sp. nov.**
–	Median lobe of aedeagus with large rectangular apical portion, apex blunt, without protrusion, but with two large acute projections preapically; apex of paramere rounded (Ôhira 1972: fig. 3)	***P.formosana***

## ﻿Discussion

In China, the tribe Dimini is represented not only by the lineages with flying species, but also by flightless ones, such as those from genera *Dima* Charpentier, 1825, *Neodima* Schimmel & Platia, 1992, and *Sinodima* Kundrata, Sormova & Qiu, 2019 (Qiu et al. 2018; Kundrata et al. 2019a, 2019b). Most of these flightless species are known from the western mountains of China (12 spp. of *Dima* and four spp. of *Neodima*) (Qiu et al. 2018, 2020; Ruan et al. 2018; Kundrata et al. 2019b; Qiu 2021), with very few species from central (one sp. of *Dima* and one sp. of *Sinodima*) and eastern China (two spp. of *Dima*) (Suzuki 1979; Qiu et al. 2018; Ruan et al. 2018; Kundrata et al. 2019a). Previously only one flightless species of Dimini was formally reported by Suzuki (1979) from Taiwan Island, i.e., *Dimanebriomorpha* Suzuki, 1979. The two flightless Dimini species from Taiwan described in this paper are morphologically similar to but also readily distinguishable from *Pseudocsikiaformosana*. The original description of *P.formosana* did not reveal whether it has reduced hind wings or not (Ôhira 1972), but based on the similarity of the elytral humeri and metaventrite between *P.formosana* and the two new species, we suppose that *P.formosana* also lacks or has reduced hind wings. The most notable characters supporting this hypothesis are the globose elytra and abdomen, the rounded elytra shoulders, and a relatively short metaventrite. These all are typical characters for the flightless species in Coleoptera (Smith 1964). The discovery of two more non-flying species in Taiwan indicates that the diverse flightless Dimini may be present not only on mainland East Asia but also on islands.

## Supplementary Material

XML Treatment for Pseudocsikia

XML Treatment for Pseudocsikia choui

XML Treatment for Pseudocsikia chanjuan

XML Treatment for Pseudocsikia formosana
